# Risk Factors, Prevention, and Primary and Secondary Management of Sciatica: An Updated Overview

**DOI:** 10.7759/cureus.31405

**Published:** 2022-11-12

**Authors:** Maryam Fairag, Raghad Kurdi, Abdullah Alkathiry, Nawaf Alghamdi, Rahaf Alshehri, Faris O Alturkistany, Abdullah Almutairi, Mohammad Mansory, Mohammed Alhamed, Ahmed Alzahrani, Abdulhadi Alhazmi

**Affiliations:** 1 Family Medicine, Makkah Healthcare Cluster, Makkah, SAU; 2 College of Medicine, Alrayan Medical College, Madinah, SAU; 3 College of Medicine, King Abdulaziz University, Rabigh, SAU; 4 General Practice, Prince Meshari Bin Saud Hospital, Baljurashi, SAU; 5 College of Medicine, Ibn Sina National College for Medical Studies, Jeddah, SAU; 6 Internal Medicine, Eradah Complex for Mental Health, Jeddah, SAU; 7 College of Medicine, King Abdulaziz University, Jeddah, SAU; 8 College of Medicine, King Saud Bin Abdulaziz University for Health Sciences, Jeddah, SAU

**Keywords:** radicular pain, complication, treatment, diagnosis, pathophysiology, sciatica

## Abstract

Sciatica is a chronic condition causing crippling low back pain radiating down to the sciatic nerve innervation area, which is the posterior thigh. It remains a major public health problem worldwide with significant socio-economic, physical, and psychological impacts. Studies suggested different diagnostic methods due to the lack of consensus on diagnostic and treatment guidelines. When it comes to the management and treatment, there is ambiguous evidence about the use of painkillers, surgical interventions, and alternative options and their effectiveness, with most studies contrasting one another in addition to the lack of high-quality trials. This review presents the available data on the current understanding of sciatica covering clinical manifestations, diagnosis and treatment modalities, prognosis, and complications since a disagreement is observed in the scientific community regarding sciatica, starting with a definition of sciatica, its epidemiological characteristics, to the management and treatment. Our review would help raise knowledge and awareness about sciatica in the health professional community and the general public since the prevalence of low back pain is high in most parts of the world and there is insufficient knowledge of sciatica in the literature.

## Introduction and background

Sciatica is a crippling condition caused by sciatic nerve root (L4-S3) compression caused by vertebral canal disk narrowing or trauma; in other words, vertebral disk protrusion or prolapse [1]. Furthermore, among the most common etiologies of sciatica is herniation due to trauma, or neural foramina stenosis. Sciatica is a clinical condition characterized by radicular pain beginning from the low back region and radiating downward along the course of the sciatic nerve. It can occur with or without lower extremity pain unilaterally or bilaterally, which may cause neurological deficits, including muscle weakness, absence of tendon reflexes or sensory deficit, numbness, and bladder dysfunction in some cases [2,3]. Sciatica is considered a major health issue worldwide and has a major impact on medical expenses as it is estimated to account annually for € 500 million of direct cost and almost € 4 billion of indirect cost in the United Kingdom, In addition to significant disability and work absenteeism worldwide [2-4].

Patients with sciatica complain of chronic pain exacerbated by lumbar spine flexion and relieved by analgesics, such as non-steroidal anti-inflammatory drugs (NSAIDs). Sciatica affects both genders, and its incidence peaks in the fourth decade of age, with an annual incidence of 1-5% [4]. It rarely occurs before 20 years of age unless it is secondary to trauma and is triggered by physical activities and frequent workplace postures [4,5]. Compression of the spinal nerve root can lead to local edema, ischemia, and, consequently, inflammation that may cause leakage from the degenerated intervertebral discs. Sciatica's common features are tenderness to pressure, muscle weakness, reduced sensation, and pain aggravated by movement [6,7].

Recent studies found that the prevalence of sciatic complaints is widely variable, ranging from 1.6% to 43%, and argued that the term 'sciatica' referring to L1-L4 nerve roots might contribute to misinterpretation of lower back pain radiating down to the leg [8]. When sciatica was defined in terms of pain distribution and/or duration, the prevalence rate was lower, and sciatica remained more prevalent in working populations with physically demanding jobs [8,9]. There is a scarcity of data on sciatica in Saudi Arabia, highlighting the need for more studies exploring sciatica among the Saudi population since the prevalence of low back pain is high (63.8- 89%) [10,11]. One study evaluating sciatica prevalence in Saudi Arabia found it to be 13.5%; the factors connected with high prevalence were found to be the 35-49 years age group, Saudi nationality, having a bachelor's degree, and being a nonhealthcare worker [11,12]. Moreover, the knowledge about sciatica was reported inadequate in Saudi Arabia despite good attitude [12]. This review presents available knowledge about sciatica, including clinical manifestations, diagnosis and treatment modalities, prognosis, and complications.

## Review

Clinical manifestations and diagnosis of sciatica

Some symptoms and signs suggesting sciatica include unilateral leg pain that is more severe than low back pain, pain radiating most commonly posteriorly at the leg and below the knee, paresthesia, and/or numbness in the involved lower leg [4]. People with sciatica usually have aching pain described as a sharp leg pain radiating below the knee and into the foot and toes, and most people report low back pain coexistence. The pain might be of sudden or slow onset and differs in severity. Numbness or tingling and loss of muscle strength in the same leg are other suggestive symptoms of nerve root involvement. The diagnosis of sciatica is mainly by clinical diagnosis based on the symptoms and findings on the examination of the patient [13]. 

A previous history should be gained from the patient whose leg pain is worse than back or knee pain since it can be a red flag for sciatica. Moreover, inquiring about the onset and distribution of pain and associated symptoms such as tingling sensation, numbness, or muscle weakness in the legs must be done [8]. Currently, there is no specific type of tests for sciatica, and doctors must do a lot of similar tests and examinations to reach a conclusive diagnosis [14]. Neural tension tests, such as the femoral nerve, slump, or straight leg raise tests, are used to confirm the sciatica diagnosis [15]. These tests can also identify neurological deficits associated with the involved nerve root (sensory deficit/absence of tendon reflexes/muscle weakness) [8]. In order to predict sciatica brought on by lumbar disc herniation, recent cohort research established clinical criteria consisting of a positive straight leg raise test at 60° (or femoral stretch test), unilateral motor weakness, mono-radicular distribution of pain, unilateral leg discomfort, and asymmetric ankle reflex [14]. Most clinical practice recommendations advise against routine imaging in patients with non-specific low back pain, either with or without sciatica, to avoid unnecessary tests, referrals, intervention, and increased costs. However, imaging is considered if the progression of symptoms takes more than 12 weeks or if the person has worsening pain or progressive neurological deficits [16]. 

Risk factors and prevention

Risk factors for sciatica can be aggregated into categories, including demographic characteristics of the individual, such as age and gender; physical stress on the spine (e.g., regular lifting and whole-body vibration); poor general health (e.g., smoking and obesity, history of lower back pain), and psychological stress (e.g., monotonous work and depression) [13]. Furthermore, risk factors can also be grouped into modifiable and non-modifiable factors [3,17,18]. Modifiable factors include smoking, obesity, and occupational factors, such as jobs requiring prolonged standing and bending, heavy manual labor, heavy lifting, and health status [3,17]. Non-modifiable factors included age, gender, and socioeconomic class [18]. A systematic review involving eight articles found that most reported risk factors were modifiable and associated with an unhealthy lifestyle [18]. Since factors are modifiable, interventions are feasible indicating that proper prevention measures targeting modifiable factors would be effective against sciatica. Prevention of sciatica primarily involves lifestyle changes, such as walking, cycling, quitting smoking, and management of the pathological cause, which can be classified as skeletal and non-skeletal causes; skeletal causes include, but are not limited to, disc herniation, degenerative changes, congenital abnormalities, and rare causes such as root avulsion that may occur with sacroiliac joint fractures of pubic rami diastases. Non-skeletal causes include infection, inflammation, neoplasm, and vascular disease [7]. Disk rupture and degenerative spine disease are more common than all non-spinal causes. Both smoking and obesity increase the risk of hospitalization for sciatica while walking or cycling to work reduces the risk [13]. Therefore, walking and cycling, which can help control weight and quit smoking, are recommended to prevent sciatica in the general population [7]. Moreover, studies have indicated that smoking cessation could lead to a 40% prevention success rate [19], while most smokers had more severe sciatica symptoms [20]. Weight control was also reported among primary care measures for sciatica, while another study found that obesity was associated with poor outcomes [21]. When studying the impact of addressing occupational factors, it was found that good outcomes were associated with modification of the working environment, tasks, and education [18,22].

Treatment

Sciatica treatment modality involves pharmacological and non-pharmacological options. However, using pain-relieving medications, such as NSAIDs for sciatica, has uncertain benefits and might have adverse effects. A systematic review of 23 articles found that active treatment was not favored over placebo and the active treatment benefits were only short-term [23]. This systemic review reported some short-term benefits from an anticonvulsant such as gabapentin for chronic sciatica. However, when compared to the placebo treatment, the adverse effect median rate of gabapentin was 17% (range: 10-30%) compared to 11% (range: 3-23%) for the placebo.

Acetaminophen Versus Placebo 

Although most painkillers for sciatica have been studied in different randomized controlled trials, most studies available have a low to moderate quality. Comparing them is still challenging since each has different populations, methods, and outcomes. Therefore, there is a lack of randomized placebo-controlled trials evaluating the efficacy of acetaminophen for sciatica [24]. However, one study found that morphine was more effective in sciatica than acetaminophen [25].

Non-Steroidal Anti-Inflammatory Drugs (NSAIDs)

A systemic review involving 10 trials shows that the use of NSAIDs in sciatica was not significantly more effective than the placebo in reducing pain or disability and some improvements resulting from NSAIDs were short-term (three weeks) [26]. It should be noted that the quality of evidence overall using the Grading of Recommendations, Assessment, Development, and Evaluations (GRADE) approach for these outcomes is low to very low. In this review, evidence from four trials suggested an increased risk of adverse effects of NSAIDs compared to placebo, aligning with another previous study [24]. Compared to the placebo, NSAIDs had 1.14 odds of adverse effects and there was no significant evidence of more NSAIDs effectiveness than the placebo [26]. Most of these adverse effects reported were mild and included headache, abdominal pain, gastrointestinal problems, and dizziness [26]. 

Though some studies evaluated the effectiveness of NSAIDs in terms of pain relief, general improvement, and adverse effects, sciatica patients found no improvement in disability [23,24,26]. A more recent study found NSAIDs more effective in pain reduction and disability improvement for short-term than placebo in patients with low back pain [27].

Systemic Corticosteroids

Moderate quality evidence from a meta-analysis of two trials in 2012 favored corticosteroids over placebo in reducing pain at short follow-up time [23]. Another trial with moderate bias risk reported pain relief at 24 hours but not at six weeks [28]. However, a large trial with a low risk of bias shows no improvement in pain, although there was a small reduction in disability [29]. Treating sciatica with opioids, antidepressants, and benzodiazepines is not recommended, and the evidence regarding these drugs is limited [24].

Epidural Injections

In people with acute, severe sciatica, the National Institute for Health and Care Excellence (NICE) guidelines recommend the use of epidural injection with local anesthesia and steroids in the lumbar nerve root area; otherwise, they would be considered for surgery [30]. A controlled trial on the use of transforaminal epidural corticosteroid injections showed findings in favor of epidural corticosteroid injections for acute sciatica patients over painkillers. However, they recommended the use of painkillers in the case of unsuccessful injection outcomes [31].

Physiotherapy Treatment 

The Dutch guidelines for general practitioners recommend exercise therapy in patients with greater than six to eight weeks of complaints that were not improved during this period [32]. Though exercise reduces pain intensity in the short term, there was no specific recommendation for a specific type of exercise apart from supervised exercise therapy, including strength exercise, nerve mobilization, and directional exercises. 

Spinal manual therapy (SMT) aims to move one or more joints within the normal range of motion for improvement of spinal joint function or motion and can be used along with exercises. Based on data from systematic reviews and meta-analysis, the Danish National Clinical Guidelines recommends using SMT together with supervised exercise and patient education. However, they advise against acupuncture, electrotherapies, and traction in general patients with sciatica and back pain [33]. 

Surgical Management of Sciatica

Surgical management of sciatica is usually considered when symptoms are persistent despite non-surgical treatment options. However, there are a few cases where surgery may be the first-line intervention. For example, these cases are cauda equina syndrome, bilateral sciatica, and tumors or severe lumbar spine fractures in addition to sciatica [34]. Sciatica can have various causes and some of which might be preferably treated with surgical interventions if feasible and indicated [1]. One of the leading causes of sciatica is lumbar disc herniation (LDH), and lumbar discectomy was found to be the surgery of choice in this case [35]. A randomized controlled trial involving patients with LDH at L4-L5 and L5-S1 for 4-12 months who underwent surgical intervention in the form of discectomy found that they had less leg pain after six months compared to patients treated conservatively [36]. Furthermore, according to another study published in 2013, patients who had to wait less than three months before undergoing discectomy had relatively less pain in the six months following the surgery than those who had their surgery delayed more than three months [37]. 

Another common cause of sciatica is lumbar spinal stenosis, for which vertebral decompression is done in cases of conservative treatment failure [38]. The gold standard technique used to improve function, relieve pain and reduce or prevent neurological deficit is open laminectomy or laminotomy. The segment and location requiring decompression depend on imaging findings and the presence of severe symptoms and radicular involvement associated with dermatome sensorial and motor changes [38,39]. There are currently controversies and discussions about the short-term and long-term results of open techniques compared to minimally invasive techniques. It was found that the unilateral laminectomy technique for minimally invasive bilateral decompression resulted in less blood loss and shorter hospital stay than the open technique; however, they both had the same complications and long-term outcomes [38].

Conservative and Alternative Treatment

The severity of sciatica symptoms can vary widely, sometimes resulting in absence from work or severely limited daily activities. To choose the best treatment strategy, it is important to consider patient preferences. Initial treatment is conservative unless the patient develops symptoms that need urgent surgery. Imaging is obtained, and additional spinal injections or surgery referrals may be considered for persistent complaints or worsening neurologic deficits [40]. The initial course of treatment focuses on pain management and maintaining function, reassuring patients that symptoms usually diminish over time, and educating them about sciatica's natural course. In addition to avoiding aggravating activities and prolonged sitting or standing and regular light exercises such as walking, swimming, or aqua therapy, patients should watch for any changes in their symptoms, such as worsening leg pain or neurological deficits, and to report them [40].

Due to recognized side effects and limited evidence on the effectiveness of painkillers for sciatica [20], pain medications should be used for a short period of time and at the lowest dose possible. Non-steroidal anti-inflammatory drugs (NSAIDs) and systemic corticosteroids are shown to have limited positive effects [41]. Both NSAIDs and corticosteroids generally have smaller effects and more adverse events than placebos [24,41,42]. Benzodiazepines, opioids, or antidepressants are not recommended since the evidence available is limited and only comes from one trial. As mentioned above, since paracetamol has no known effects on sciatica and no placebo-controlled trials were identified, it is also not recommended [24,42].

The recommendation of lumbar nerve root extraforaminal steroidal injections is controversial since the beneficial effect is thought to be relatively minimal and temporary [42]. However, the NICE recommends that epidural injection of local anesthetic and steroids be considered in acute and severe sciatica [30,42]. Clinical guidelines recommend referring patients for surgical evaluation if they have persistent pain after receiving conservative treatment for 12 weeks, with imaging supporting clinical indications and showing lumbar disc herniation at the nerve root level [42].

The most common surgical procedure is an open micro-discectomy, which may or may not involve using a surgical microscope or other magnification equipment, in addition to minimally invasive surgical techniques like endoscopic surgery are also available options [42,43]. 

When comparing surgical management to conservative care for sciatica patients, a systematic review reported low-quality evidence that shorter-term leg pain relief from surgery followed by six weeks of conservative care had better outcomes than pain relief from longer-term conservative management [42]. They also reported no differences between surgery and conservative treatment on any clinical outcomes at the one- and two-year follow-ups.

Prognosis and complications

If left untreated, most cases of sciatica resolve themselves in less than four to six weeks without any long-term complications. The patient may have a prolonged recovery in more severe cases or in cases when the neurologic deficit is present, but recovery is still excellent. According to some studies, having chronic, recurrent sciatica increases the risk of poor occupational mechanics, psychological depression, and poor socio-economic situations [15]. 

Analyzing the treatment outcomes of sciatica patients is difficult because every study has a different set of parameters to determine success; thus, the results are either misinterpreted or hyped. Patients with chronic pain (lasting more than six months) typically experience worse outcomes from surgery than patients with acute pain (less than six months) [44]. Some studies have reported a cure rate of more than 75%, while others reported cure rates of less than 50% from surgical interventions. Several more recent orthopedic techniques exist to manage sciatica, with short-term success rates of at least 70% [13,15]. Irrespective of the short-term outcome, most sciatica patients experience persistent or recurrent pain in the long run, are dependent on pain medications, are disabled, and have a poor quality of life [15].

If the pressure on the sciatic nerve is not relieved, untreated sciatic nerve compression result in complications, such as progressively worsening pain, loss of muscular strength in the affected leg, loss of bowel and/or bladder function, permanent nerve damage, persistent muscular weakness, including drop foot, paresthesia, and hyperalgesia [45]. Even though it's uncommon, sciatica could cause cauda equina syndrome, which is caused by compression of the cauda equina, and this should be treated immediately [34]. If prompt treatment is not received, sciatica can lead to permanent disability involving difficulty walking, lower-body paralysis, and also sexual dysfunction [34,45]. 

## Conclusions

Though most cases of sciatica are spontaneously resolved without any treatment, it is still a debilitating health problem with a high prevalence rate. Lifestyle change for healthy living is recommended to reduce the burden posed by sciatica, including changing modifiable risk factors, namely: smoking, obesity, prolonged standing and bending forward, heavy manual labor, and heavy lifting. Walking, cycling, weight control, and physical exercise have also been shown to be effective against sciatica. When these conservative treatment methods are unsuccessful, surgical intervention is recommended to relieve pain for a short period. However, no sufficient data indicates any surgical method association with better long-term outcomes than conservative management. Therefore, surgery remains the first-line intervention for sciatica coupled with surgical pathologies such as disc herniation, cauda equina syndrome, rumors, and severe fractures. Although this paper attempted to provide an overview of sciatica prevention and treatment, extensive studies are recommended to explore sciatica further and shed light on the unknown.
